# Anatomical-related factors and outcome of percutaneous short-term spinal cord stimulation electrode shift in patients with disorders of consciousness: a retrospective study

**DOI:** 10.3389/fnagi.2024.1403156

**Published:** 2024-07-02

**Authors:** Qiheng He, Chaozhi Yang, Yangxi Xu, Hongchuan Niu, Haitao Wu, Haitao Huang, Xiaoke Chai, Tianqing Cao, Nan Wang, Peiling Wong, Jianghong He, Yi Yang, Jizong Zhao

**Affiliations:** ^1^Brain Computer Interface Transitional Research Center, Beijing Tiantan Hospital, Capital Medical University, Beijing, China; ^2^Department of Neurosurgery, Affiliated Hospital of Zunyi Medical University, Zunyi, Guizhou, China; ^3^Department of Neurosurgery, The People’s Hospital of Liaoning Province, Shengyang, China; ^4^Department of Neurosurgery, Peking University International Hospital, Beijing, China; ^5^China National Center for Neurological Disorders, Beijing, China; ^6^Department of Neurosurgery, Beijing Tiantan Hospital, Capital Medical University, Beijing, China; ^7^Department of Physical Therapy and Assistive Technology, National Yang Ming Chiao Tung University, Hsinchu, Taiwan; ^8^Chinese Institute for Brain Research, Beijing, China; ^9^Beijing Institute of Brain Disorders, Beijing, China; ^10^China National Clinical Research Center for Neurological Diseases, Beijing, China

**Keywords:** spinal cord stimulation, electrode shift, disorders of consciousness, outcome, anatomical factor, percutaneous, short-term

## Abstract

**Background:**

Disorders of consciousness (DoC) represent a spectrum of neurological conditions that pose significant treatment challenges. Percutaneous short-term spinal cord stimulation (SCS) has emerged as a promising experimental diagnostic treatment to assess and potentially improve consciousness levels. However, the effectiveness of this intervention is frequently compromised by the shift of electrodes, particularly in the cervical region, which can negatively affect therapeutic outcomes.

**Methods:**

This retrospective study aimed to study if electrodes shift in percutaneous short-term SCS in patients with DoC would affect the outcome. We analyzed the relationship between electrode shift length and patient outcome, as well as the correlation with various anatomical parameters, including the actual length of the cervical spine, linear length, spinal canal transverse diameter, spinal canal diameter, and C2 cone height, in a cohort of patients undergoing the procedure.

**Results:**

Our findings revealed that in patients with better outcome, there are significant less patient with electrode shift (*p* = 0.019). Further, a linear correlation was found between the length of electrode shift and patients’ outcome (Rho = 0.583, *p* = 0.002), with longer shift lengths associated with poorer outcomes. Contrary to our expectations, there was no significant association between the measured anatomical parameters and the extent of electrode shift. However, a trend was found between the actual length of the cervical spine and the shift of the electrode (*p* = 0.098). Notably, the shorter spinal canal transverse diameter was found to be significantly associated with better outcome in patients with DoC receiving percutaneous short-term SCS (*p* = 0.033).

**Conclusion:**

These results highlight the clinical importance of electrode stability in the cervical region during SCS treatment for patients with DoC. Ensuring secure placement of electrodes may play a crucial role in enhancing patients’ outcome and minimize postoperative complications. Given the lack of association with expected anatomical parameters, future research should investigate other factors that could impact electrode stability to optimize this therapeutic intervention.

## 1 Introduction

Disorders of consciousness (DoC) represent a wide spectrum of neurological conditions, which include coma, vegetative state/unresponsive wakefulness state (*VS*/UWS), and minimally conscious state (MCS), posing profound challenges to medical management and patient rehabilitation (Eapen et al., 2017; Giacino et al., 2018). These conditions result from significant brain injuries and are characterized by varied levels of wakefulness and awareness (Edlow et al., 2021). Traditional treatment modalities are often limited in their effectiveness, and only amantadine is considered as an effective drug through randomized controlled trial (Giacino et al., 2012; Ruhl et al., 2022). Thus, clinicians are seeking alternative therapeutic avenues to promote the recovery of patients with disorders of consciousness. Among these, neuromodulation techniques offer a novel approach to facilitating changes in the patients’ brain activity patterns in hopes of restoring consciousness (Vanhoecke and Hariz, 2017; Wu et al., 2023). This method represents an emerging frontier in the treatment of disorders of consciousness, attempting to bridge the gap between the damaged neuronal pathways and the complex network that governs wakefulness and awareness (Raguz et al., 2021; Cao et al., 2023).

Spinal cord stimulation (SCS) is an innovative therapeutic intervention for DoC that targets the neural structures implicated in arousal and awakening by delivering electrical impulses directly to the spinal cord (Martens et al., 2017; Yamamoto et al., 2017). This modality of treatment demonstrates potential not only for transient cognitive improvements but also for facilitating long-term recovery in brain function (Yang et al., 2022). Recently, percutaneous short-term SCS has been reported to may be useful in the treatment of DoC (Yang et al., 2022; Huang et al., 2023). This minimally invasive approach affords clinicians the ability to directly engage with neuronal circuits that may be responsible for the modulation of conscious states (Zhuang et al., 2023). This treatment method implants electrodes into the body for a short time and is currently mainly used for diagnostic treatment. Therefore, this requires the electrode to be effective within a short period of time, so the requirement for full effectiveness is more important. However, whether anatomical-related factors will affect the effectiveness of percutaneous short-term SCS in patients with DoC to restore consciousness remain unknown.

Recognizing the importance of these issues, the present study aims to investigate the effectiveness of percutaneous short-term SCS in treating DoC and the possible anatomical factors related to electrode shift which could affect patients’ outcome. Through a meticulous analysis of various anatomical parameters and their relationship to electrode shift, this research seeks to provide valuable insights into improving the stability of SCS implantation process and, ultimately, the outcomes of patients with DoC.

## 2 Materials and methods

### 2.1 Study subjects

This study retrospectively analyzed patients who underwent percutaneous short-term SCS implantation surgery in the department of neurosurgery, Beijing Tiantan Hospital from January 2021 to December 2021. The inclusion criteria were: (1) Meet the diagnosis of prolonged DoC according to EAN guideline (Kondziella et al., 2020). (2) The patients’ neurological function showed no significant progress 3 months before surgery. (3) Postoperative CT scan can be extracted. (4) Informed consent has been obtained from patient’s legal representative or caregiver. The exclusion criteria were: (1) Significant changes in consciousness within 4 weeks. (2) Serious complications or surgical contraindications. (3) No postoperative electrode shifts. Finally, 25 patients were enrolled in this study. The study was approved by the ethics committee of Beijing Tiantan Hospital, Capital Medical University (KYSQ 2020-387-01).

### 2.2 Data collection

**Figure 1:**
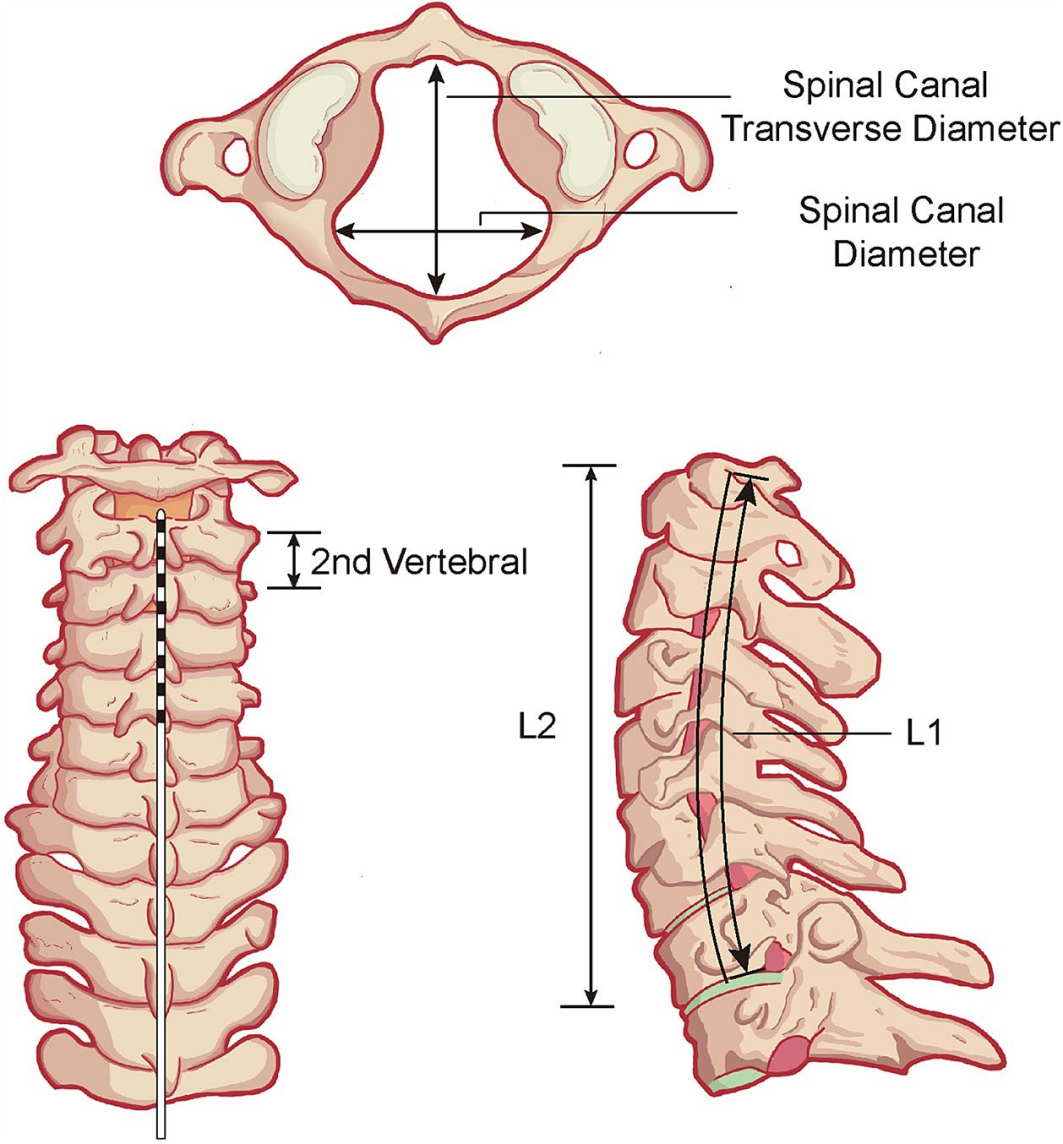
Schematic diagram of anatomical parameters. L1, the actual length of the cervical spine. L2, the straight length from the uppermost edge to the lowermost edge of the cervical spine.

The baseline variables including age, sex, pathogeny, duration, and preoperative JFK Coma Recovery Scale-Revised (CRS-R) were extracted from electronic medical record system (Giacino et al., 2004). The patients’ postoperative DICOM format CT images are imported into the Bee DICOM Viewer (V3.1.1) software and measured using built-in measurement tools. All measurements were independently completed by two senior doctors to ensure accuracy. Taking the upper edge of the first cervical vertebra pyramid as the zero point, the value of the intraoperative electrode site D1 is defined as the difference between the upper edge of the intraoperative electrode site and the zero point, and the postoperative electrode site D2 is defined as the difference between the upper edge of the postoperative electrode site and the zero point. The length of electrode shift is defined as the difference between D2 and D1. The actual length of the cervical spine L1 is defined as the curve length passing through each cervical vertebra body, and the straight length of the cervical spine L2 is defined as the straight length from the uppermost edge to the lowermost edge of the cervical spine (Figure 1). Spinal canal transverse diameter, spinal canal diameter, and second cervical vertebral body height were also collected. As for postoperative data, except for postoperative CRS-R at follow-up, whether patients developed paroxysmal sympathetic hyperexcitability (PSH) was also collected. Patients whose CRS-R scores increased by more than 2 points after surgery were considered to be in the good outcome group; those whose scores improved less than 2 points were considered to be in the fair outcome group; and those whose scores decreased were considered to be in the poor outcome group.

### 2.3 Percutaneous short-term SCS implantation surgery

After general anesthesia, the patient was placed in a prone position under fluoroscopy, and the T7/8 intervertebral space was positioned under C-arm as the puncture point. The electrode (#3777, Medtronic, USA) was placed in the sublaminar epidural space at the level of C2 and secured. Postoperatively, measures such as keeping the cervical collar fixed, turning along the axis when turning, and preventing neck flexion were taken to prevent the electrode from falling off as much as possible. The day after the st-SCS operation, electric stimulation was applied to the patient’s dorsal column with a voltage of 2.5 V, and a frequency of 70 Hz with 120us wave width. The stimulation was performed in 15-min on/15-min off cycles from 8 AM to 8 PM. The overall stimulation lasted for 14 days and then the electrode was removed.

### 2.4 Statistical analysis

SPSS 27 (IBM, USA) software was used for data description and statistical analysis. Chi-square test was used to examine categorical data. For continuous variables, Shapiro–Wilk test was used to determine the normality. Mean ± standard deviation or median (IQR) were separately used to describe normally distributed or non-normally distributed variables. According to the variance, student *t*-test or Mann Whitney test were used to perform statistical analysis. *p* < 0.05 was considered to be significantly different.

## 3 Results

### 3.1 Study population

25 patients with DoC undergoing percutaneous short-term SCS surgery were included in this study. According to the degree of electrode shift, patients were categorized as less electrode shift group (*N* = 13) and more electrode shift group (*N* = 12) (Table 1). In this cohort, 15 (60.0%) patients were male with the mean age of 43.5 ± 15.7 years. We found no significant difference in age (*t*-test, *p* = 0.269) or sex (chi-square test, *p* = 0.327) between more electrode shift group and less electrode shift group. Regarding pathogeny, there are separately 5 (38.5%) and 2 (16.7%) patients with DoC caused by trauma in less electrode shift group and more electrode shift group with no significant difference (chi-square test, *p* = 0.090). As for the duration of DoC, no significant difference was found between less electrode shift group (5.8 ± 2.5 months) and more electrode shift group (6.0 ± 3.1 months) (*t*-test, *p* = 0.840). Regarding the diagnosis at admission, there are 6 (46.2%) patients with MCS in less electrode shift group and 7 (58.3%) patients with MCS in more electrode shift group separately, and no significant difference was found (chi-square test, *p* = 0.543). Regarding their preoperative CRS-R, the score of patients in less electrode shift group (7 [5–9]) is of no difference with that of more electrode shift group (8 [6–9], Mann–Whitney test, *p* = 0.470). The results suggested no significant differences in baseline information between the two groups.

**Table 1:** Baseline characteristics in patients with DoC undergoing percutaneous short-term SCS.

Variables	All patients (*N* = 25)	Less shift group (*N* = 13)	More shift group (*N* = 12)	*p* value
Age, y, mean (SD)	43.5 ± 15.7	40.1 ± 15.2	47.2 ± 16.2	0.269
Sex, male, *n* (%)	15 (60.0)	9 (69.2)	6 (50.0)	0.327
Pathogeny, *n* (%)				0.090
Anoxia	7 (28.0)	5 (38.5)	2 (16.7)	
Stroke	11 (44.0)	3 (23.1)	8 (66.7)	
Trauma	7 (28.0)	5 (38.5)	2 (16.7)	
Duration, months, *n* (%)				0.582
< 6 months	13 (52.0)	7 (53.8)	6 (50.0)	
≥ 6 months	12 (48.0)	6 (46.2)	6 (50.0)	
Diagnosis, *n* (%)				0.543
*VS*/UWS	12 (48.0)	7 (53.8)	5 (41.7)	
MCS	13 (52.0)	6 (46.2)	7 (58.3)	
Preoperative CRS-R, point, median (IQR)	8 (6–9)	7 (5–9)	8 (6–9)	0.470

DoC, Disorders of consciousness; SCS, spinal cord stimulation; VS/UWS, vegetative state/unresponsive wakeness state; MCS, minimally conscious state; CRS-R, coma recovery scale-revised.*p* < 0.05, statically different.

### 3.2 Potential factors related to percutaneous short-term SCS electrode shift

To explore if anatomical factors are related to the electrode shift in patients with DoC, we primarily analyzed the relationship between cervical-related anatomical parameters and electrode shift. The results suggest that although D1 (the difference between the upper edge of the intraoperative electrode site and the zero point, *t*-test, *p* = 0.510) and D2 (the difference between the upper edge of the postoperative electrode site and the zero point, *t*-test, *p* = 0.083) are of no difference between groups, the length of shift (D2-D1) is significantly larger in more electrode shift group than less electrode shift group (Mann–Whitney test, *p* < 0.001). Then we explored if actual length of the cervical spine (L1) or the straight length of the cervical spine (L2) are related to the electrode shift. We found no significant difference in L2 and electrode shift (Mann–Whitney test, *p* = 0.110), but it seems to be larger L1 in less electrode shift group than more electrode shift group, with the potential to be significant (Mann–Whitney test, *p* = 0.098). Similarly, no difference was found in L1-L2 or L1/L2 with the relationship of electrode shift. The results suggest that it seems that the larger the actual length of the cervical spine (L1), the less opportunities to have electrode shift after surgery.

Then we analyzed the relationship of spinal cord diameter and spinal canal transverse diameter with the relationship with electrode shift, however, no significant difference was found. We also explored if C2 vertebral body height would be larger in less electrode shift group. Consistently, no significant difference was found (*p* = 0.815) (Table 2).

**Table 2:** Potential factors related to percutaneous short-term SCS electrode shift.

Variables	All patients (*N* = 25)	Less shift group (*N* = 13)	More shift group (*N* = 12)	*p* value
T1, mm, mean (SD)	22.51 ± 9.51	23.75 ± 10.00	21.17 ± 9.18	0.510
T2, mm, mean (SD)	29.53 ± 10.12	26.17 ± 10.26	33.18 ± 8.98	0.083
Length of shift (T2-T1), mm, median (IQR)	4.38 (2.00–11.19)	2.06 (1.81–3.27)	11.19 (6.50–18.87)	< 0.001
Actual length of the cervical spine (L1), mm, median (IQR)	120.85 (115.98–130.03)	125.97 (118.30–131.72)	117.88 (113.33–124.32)	0.098
Straight length of the cervical spine (L2), mm, median (IQR)	118.82 (113.15–128.03)	122.72 (117.17–128.72)	115.97 (111.63–122.59)	0.110
Difference in L1 and L2 (L1-L2), mm, mean (SD)	2.23 ± 1.41	2.23 ± 1.37	2.23 ± 1.52	0.996
Ratio of L1 to L2 (L1/L2), mean (SD)	1.02 ± 0.01	1.02 ± 0.01	1.02 ± 0.01	0.779
spinal canal diameter, mm, mean (SD)	24.20 ± 3.26	24.84 ± 1.88	23.51 ± 4.28	0.319
spinal canal transverse diameter, mm, median (IQR)	18.36 (16.40–19.57)	18.04 (15.84–19.18)	19.09 (18.14–20.56)	0.205
C2 vertebral body height, mm, mean (SD)	33.31 ± 3.36	33.47 ± 2.75	33.15 ± 4.04	0.815

DoC, Disorders of consciousness. SCS, spinal cord stimulation.*p* < 0.05, statically different.

### 3.3 The relationship between anatomical factors and outcome in patients with DoC receiving percutaneous short-term SCS

**Figure 2:**
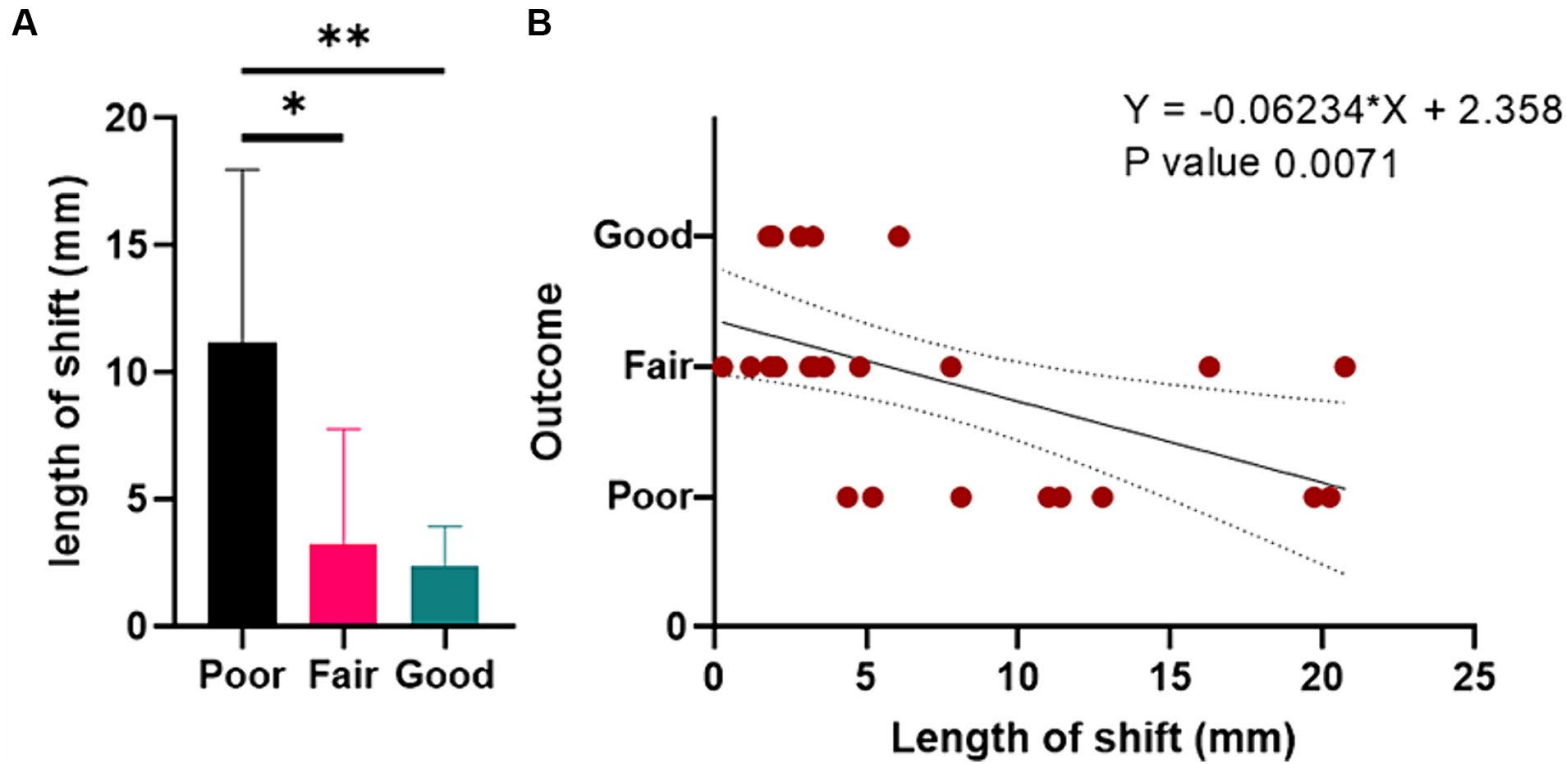
The relationship between the length of shift and outcome in patients with DoC receiving percutaneous short-term SCS. **(A)** Bar graph. **(B)** The linear correlation between the length of shift and outcome. DoC, disorders of consciousness. SCS, spinal cord stimulation.

To explore if electrode shift would affect the outcome of patients with DoC receiving percutaneous short-term SCS, we analyzed if anatomical factors are associated with the outcome. We found in patients with better outcome, there are significant less patient with electrode shift (*p* = 0.019). Further, the outcome of patients gradually increased as the length of electrode shift decreased with a significant difference (Figure 2A, *p* = 0.012). Through correlation analysis, we also found that patients’ outcome was linearly related to the length of electrode shift (Figure 2B, Spearman correlation, Rho = 0.583, *p* = 0.002) (Table 3).

**Table 3:** Characteristics of DoC patients according to outcome.

Variables	Outcome			
	Poor (*N* = 8)	Fair (*N* = 11)	Good (*N* = 6)	*p* Trend
Age, y, mean (SD)	42.0 ± 16.8	48.3 ± 13.7	36.7 ± 17.6	0.345
Sex, male, *n* (%)	4 (50.0)	7 (63.6)	4 (66.7)	0.777
Pathogeny, *n* (%)				0.275
Anoxia	0 (0.0)	5 (45.5)	2 (33.3)	
Stroke	5 (62.5)	4 (36.4)	2 (33.3)	
Trauma	3 (37.5)	2 (18.2)	2 (33.3)	
Duration, months, *n* (%)				0.975
< 6 months	4 (50.0)	6 (54.5)	3 (50.0)	
≥ 6 months	4 (50.0)	5 (45.5)	3 (50.0)	
Diagnosis, *n* (%)				0.094
*VS*/UWS	6 (75.0)	5 (45.5)	1 (16.7)	
MCS	2 (25.0)	6 (54.5)	5 (83.3)	
Preoperative CRS-R, point, mean ± SD	7 (6–8)	8 (5–9)	9 (7–12)	0.203
Electrode shift, yes, no (%)	7 (87.5)	4 (36.4)	1 (16.7)	0.019
T1, mm, mean (SD)	19.64 ± 9.93	24.42 ± 7.16	22.80 ± 13.12	0.574
T2, mm, mean (SD)	31.25 ± 7.77	30.34 ± 9.95	25.76 ± 13.62	0.587
Length of shift (T2-T1), mm, median (IQR)	11.19 (6.67–16.25)	3.29 (1.95–6.28)	2.38 (1.91–3.25)	0.012
Actual length of the cervical spine (L1), mm, median (IQR)	121.05 (110.77–125.32)	120.45 (117.40–125.15)	130.38 (118.11–131.84)	0.451
Straight length of the cervical spine (L2), mm, median (IQR)	117.74 (108.85–122.74)	118.82 (115.47–123.27)	128.03 (117.17–129.28)	0.41
Difference in L1 and L2 (L1-L2), mm, mean (SD)	2.55 ± 1.65	2.21 ± 1.45	1.83 ± 1.11	0.655
Ratio of L1 to L2 (L1/L2), mean (SD)	1.02 ± 0.01	1.02 ± 0.01	1.01 ± 0.01	0.541
spinal canal diameter, mm, mean (SD)	22.87 ± 3.83	24.75 ± 3.34	24.98 ± 1.99	0.387
spinal canal transverse diameter, mm, median (IQR)	19.75 (18.74–20.88)	18.36 (17.02–18.92)	16.17 (14.84–18.29)	0.033
C2 vertebral body height, mm, mean (SD)	33.87 ± 2.53	32.59 ± 4.14	33.91 ± 2.99	0.651

DoC, Disorders of consciousness; VS/UWS, vegetative state/unresponsive wakeness state; MCS, minimally conscious state.*p* < 0.05, statically different.

For the actual length of the cervical spine, straight length of the cervical spine and their calculations, there is no significant association with patients’ outcome (*p* > 0.05). We also found no significant difference in spinal canal diameter (*t*-test, *p* = 0.387). notably, the shorter spinal canal transverse diameter was found to be significantly associated with better outcome in patients with DoC receiving percutaneous short-term SCS (Mann–Whitney test, *p* = 0.033). No significant association was found between C2 vertebral body height and patients’ outcome (*t*-test, *p* = 0.651) (Table 4).

**Table 4:** Postoperative PSH in patients with DoC undergoing percutaneours short-term SCS.

Variables	Postoperative PSH			
	No (*N* = 12)	Mild (*N* = 9)	Severe (*N* = 4)	*p* Trend
Age, y, mean (SD)	50.3 + 13.3	35.2 + 15.4	41.8 + 17.6	0.089
Sex, male, *n* (%)	6 (50.0)	5 (55.6)	4 (100.0)	0.198
Pathogeny, *n* (%)				0.022
Anoxia	5 (41.7)	1 (11.1)	1 (25.0)	
Stroke	7 (58.3)	4 (44.4)	0 (0.0)	
Trauma	0 (0.0)	4 (44.4)	3 (75.0)	
Duration, months, *n* (%)				0.965
< 6 months	6 (50.0)	5 (55.6)	2 (50.0)	
≥ 6 months	6 (50.0)	4 (44.4)	2 (50.0)	
Diagnosis, *n* (%)				0.057
*VS*/UWS	3 (25.0)	7 (77.8)	2 (50.0)	
MCS	9 (75.0)	2 (22.2)	2 (50.0)	
Preoperative CRS-R, point, mean ± SD	8 (7–10)	7 (5–8)	8 (5–14)	0.197
Electrode shift, yes, no (%)	7 (58.3)	4 (44.4)	1 (25.0)	0.495
T1, mm, mean (SD)	21.59 + 8.30	20.58 + 9.48	29.59 + 12.24	0.269
T2, mm, mean (SD)	28.59 + 12.01	29.37 + 6.69	32.70 + 12.40	0.794
Length of shift (T2-T1), mm, median (IQR)	5.43 (2.90–10.57)	4.38 (1.81–18.01)	2.66 (1.95–4.73)	0.533
Actual length of the cervical spine (L1), mm, median (IQR)	119.47 (117.18–129.94)	120.85 (110.60–125.32)	130.38 (121.38–132.49)	0.224
Straight length of the cervical spine (L2), mm, median (IQR)	117.46 (113.24–127.37)	119.33 (108.85–123.29)	128.03 (120.26–128.98)	0.322
Difference in L1 and L2 (L1-L2), mm, mean (SD)	2.48 + 1.49	1.85 + 1.36	2.33 + 1.52	0.615
Ratio of L1 to L2 (L1/L2), mean (SD)	1.02 + 0.01	1.02 + 0.01	1.02 + 0.01	0.596
spinal canal diameter, mm, mean (SD)	24.13 + 3.06	23.81 + 4.04	25.32 + 2.25	0.755
spinal canal transverse diameter, mm, median (IQR)	18.17 (15.89–18.92)	18.45 (17.02–20.49)	20.17 (15.40–21.69)	0.34
C2 vertebral body height, mm, mean (SD)	32.51 + 4.12	34.00 + 2.82	34.19 + 1.44	0.534

DoC, Disorders of consciousness; VS/UWS, vegetative state/unresponsive wakeness state; MCS, minimally conscious state; IEM, intreoperative elecphysiological monitoring.*p* < 0.05, statistically different.

### 3.4 Postoperative PSH in patients with DoC undergoing percutaneous short-term SCS

We would further like to know if there are factors that are related to postoperative PSH. According to whether patients develop PSH and the severity of PSH, they are divided into three groups: no (*N* = 12) postoperative PSH group, mild (*N* = 9) postoperative PSH group and severe (*N* = 4) postoperative PSH group. Among three groups, there is no difference in patients’ age (*p* = 0.089) and sex (*p* = 0.198). Regarding pathogeny, there are significantly more trauma cases (3, 75.0%) in the severe postoperative PSH group while 7 (58.3%) stroke cases and 5 (41.7%) anoxia cases in the no postoperative PSH group (*p* = 0.022), suggesting trauma cases are more likely to develop postoperative PSH after percutaneous short-term SCS treatment. No significant difference was found in duration among groups. Meanwhile, we found nearly significant difference in patients with MCS (9, 75.0%) in the no postoperative PSH group, suggesting they are less likely to develop PSH than patients with *VS*/MCS (*p* = 0.057). As for Preoperative CRS-R, no significant difference was found among groups (*p* = 0.197).

We also explored if electrode shift is associated with postoperative PSH. There are separately 7 (58.3%), 4 (44.4%) and 1 (25.0%) patients with electrode shift in no, mild and severe postoperative PSH group, and the length of shift seems to be decreasing with more severe PSH. However, the electrode shift or the length of shift are of no significant difference. The results suggest that with existing treatment methods, there is still a high chance of PSH occurring after implantation in the optimal position. As for the length of the cervical spine, spinal canal diameter, C2 vertebral body height and related anatomical factors, no significant difference was found among three groups (*p* > 0.05).

## 4 Discussion

The findings from this study underscore the critical issue of electrode shift in the cervical region when performing percutaneous short-term SCS in patients with DoC, which has a direct and adverse impact on the short-term therapeutic efficacy of SCS in patients with disorders of consciousness. Despite our initial hypothesis, anatomical factors such as the actual length of the cervical spine, spinal canal diameter, spinal canal transverse diameter, and the height of the C2 vertebral body were not associated with the length of electrode shift. Interestingly, we found a significant linear correlation between the length of electrode shift with the outcome in patients with DoC. The data suggests that the greater the extent of electrode shift, the worse the outcome in the patients, potentially due to decreased stimulation efficacy or increased interval of therapy disruption. This finding indicates that securing the electrode optimally is of paramount importance to improving patient outcomes.

By modulating neural activity and possibly promoting neuroplasticity, spinal cord stimulation emerges as a complementary approach capable of augmenting the limited therapeutic options currently available for patients with disorders of consciousness. Evoked compound action potentials (ECAPs) are generated when electric stimuli are applied to the dorsal columns of the spinal cord, which predominantly consist of sensory fibers that conduct information toward the brain. These action potentials propagate along the ascending sensory pathways and are thought to facilitate the activation of various subcortical structures, including the thalamus and reticular formation, which play crucial roles in modulating consciousness. The stimulation of these pathways is believed to enhance the synaptic efficacy and promote neuroplasticity, potentially leading to improvements in the neural circuits involved in consciousness. Such theoretical and empirical frameworks provide a basis for understanding how SCS might contribute to the restoration of awareness in patients with disorders of consciousness, offering hope for augmenting their responsive states. Currently, non-surgical treatments include drug treatments such as amantadine, levodopa, baclofen, and zolpidem, as well as treatments including hyperbaric oxygen therapy, repetitive transcranial magnetic stimulation (rTMS), transcranial direct current stimulation (tDCS), midline nerve stimulation (MNS), and transcutaneous vagus nerve stimulation (taVNS) (Gosseries et al., 2014; Edlow et al., 2021; Ruhl et al., 2022; Somaa et al., 2022; Vitello et al., 2023; Marino, 2024). It is also pertinent to consider the emerging evidence surrounding invasive neuromodulation techniques, notably deep brain stimulation (DBS). DBS involves the placement of electrodes in specific brain regions, which, when stimulated, can modulate neural activity at deeper levels. Studies targeting nuclei such as the thalamus, basal ganglia, and limbic structures have shown promising results in altering the state of consciousness and promoting cognitive function in DoC patients. For instance, research indicates that thalamic stimulation may help in ‘reactivating’ neural networks that are crucial for consciousness, potentially leading to significant clinical improvements in responsiveness in some patients (Yang et al., 2023). These findings underscore the potential utility of DBS alongside other methods like SCS in managing complex conditions like DoC, suggesting a multimodal approach might be the most effective in modulating the intricate neural pathways implicated in consciousness. SCS implants the stimulating electrode into the C2-C4 cervical spinal cord segment. The stimulation signal is transmitted downward from the neural network to the effect muscle group, producing an action potential, while activating the reticular structure, thalamus, dopamine, and norepinephrine nerve nuclei upward to increase cerebral blood flow and improve brain function to increase consciousness level (Mazzone et al., 1995; Visocchi et al., 2001; McDonagh et al., 2007; Liu et al., 2008; Yamamoto et al., 2012). Through the strategic placement of electrodes along the cervical spinal column, SCS plays a pivotal role in regulating wakefulness and attention. Its role in clinical practice may provide a gateway to enhanced rehabilitation strategies and improved quality of life for individuals who have suffered severe brain injuries.

After the stimulator is implanted at the ideal position of the spinal cord segment, a pulse is generated at the black dot mark to activate the dorsal column of the spinal cord and produce electrically evoked compound action potentials (ECAPs) (Foreman and Linderoth, 2012; Dietz et al., 2022). The ECAP is transmitted back through the side branch of primary Aβ input to stimulate interneurons and ultimately trigger action potentials of muscle effectors. After the stimulation electrode is turned on, if the implantation position is shifted, the generated stimulation electric field may be decreased.

There are three main aspects of complications of SCS surgery: implant-related complications, biological complications, and program or treatment-related complications. Electrode shift is the most common and serious complication associated with implant-related complications. Existing literature reports that the rate of electrode shift in surgery for failed back surgery syndrome (FBSS) and complex regional pain syndrome (CRPS) is 2.1 to 27% (Fiume et al., 1995; Kumar et al., 2006; Klase et al., 2009). When the electrode is shifted, minor adjustments to the program parameters may be required, and major adjustments may require surgical fine-tuning. Patients with severe shift may even need to have the electrode re-implanted (Eldabe et al., 2016). There are currently no reports of electrode shift in SCS surgery for patients with DoC. For SCS implantation for pain relief, the probability of electrode shift can be reduced by intraoperative awakening of patients to relieve pain (Hwang et al., 2019). However, for patients with DoC, long-term maintenance of tracheotomy and bed rest lead to impaired ventilation and gas exchange function, and intraoperative awakening seems to be impossible. Moreover, patients with DoC lack subjective expression, and clinical manifestations will be affected by abnormal muscle tone and muscle capacity of patients. Therefore, the accuracy and precision are not high.

In this study, we found percutaneous short-term SCS may be promising for patients with DoC. By stimulating specific neural pathways, there is the potential to not only awaken cognitive functions in these individuals but also to ameliorate a variety of motor and respiratory impairments, refine muscle tone, and address other related complications. This could dramatically improve the quality of life for affected patients and provide a cornerstone for longer-term rehabilitation strategies. Surgeons, neurologists, and rehabilitation specialists should take cognizance of the linear correlation between electrode loss and patient prognosis, as it underscores the critical need for secure electrode placement and monitoring to prevent electrode shift.

Despite the insights provided by this research, there are several limitations that need to be addressed. The study’s retrospective design is inherently limited by the quality and completeness of recorded data. Prospective studies are warranted to more accurately assessing the relationship between electrode shift and outcomes. Meanwhile, a non-shift group may provide further informative results, which was not included in this study. The study measured several anatomical parameters, other potential anatomical or biomechanical factors influencing electrode stability (like neck muscle tone, ligament integrity) were not analyzed. Enhancing future research with these considerations in mind could provide deeper insights and help in fine-tuning therapeutic strategies for patients undergoing SCS for DoC.

## 5 Conclusion

The study showed the importance of electrode stability in SCS for patients with DoC, and the electrode shift may lead to worse outcome in those patients. While anatomical dimensions do not seem to play a decisive role in electrode shift, the actual length of the cervical spine and its relationship to shift could present a new avenue of investigation in achieving improved patient outcomes.

## Data availability statement

The data supporting the conclusions of this article will be made available by the corresponding author, upon reasonable request.

## Ethics statement

The studies involving humans were approved by IRB of Beijing Tiantan Hospital. The studies were conducted in accordance with the local legislation and institutional requirements. The participants provided their written informed consent to participate in this study.

## Author contributions

QH: Conceptualization, Writing – original draft, Writing – review & editing. CY: Formal analysis, Writing – review & editing. YX: Data curation, Writing – review & editing. HN: Data curation, Writing – review & editing. HW: Data curation, Writing – review & editing. HH: Data curation, Writing – review & editing. XC: Data curation, Writing – review & editing. TC: Data curation, Writing – review & editing. NW: Data curation, Writing – review & editing. PW: Writing – review & editing. JH: Supervision, Writing – review & editing. YY: Funding acquisition, Project administration, Supervision, Validation, Writing – review & editing. JZ: Supervision, Writing – review & editing.
